# “You’re opening Pandora’s Box”: Public attitudes on AI and robotics in Australian agriculture

**DOI:** 10.1371/journal.pone.0332461

**Published:** 2025-09-15

**Authors:** Angie Sassano, Robert Sparrow, Christopher Mayes, Cheryl Travers, Megan Moss, Chris Degeling

**Affiliations:** 1 Department of Philosophy, Monash University, Melbourne, Australia; 2 School of Philosophy, Deakin University, Waurn Ponds, Australia; 3 Australian Centre for Health, Engagement, Evidence and Values (ACHEEV), University of Wollongong, Wollongong, Australia; Universitat de Girona, SPAIN

## Abstract

The use of artificial intelligence (AI) and robotics is widely expected to revolutionise agriculture. Although an emerging literature is bringing into conversation AI and agricultural ethics, there has been little attention paid to public attitudes regarding such technological change. Using data collected in 12 dialogue groups conducted across rural and metropolitan Australia, this paper examines public perceptions of the social and ethical impacts of AI and robotics in agriculture. We identify and map a diversity of views regarding the possible risks and benefits of AI and robotics, and the value of agriculture in the context of a future of ‘farmerless farming.’ Our results add depth and nuance to the existing, mostly quantitative, literature on public attitudes towards agricultural robotics and AI and constitute a valuable resource for policymakers, or other stakeholders who want to engage with public opinion regarding these technologies.

## 1. Introduction

Technological innovations have driven agricultural change for centuries. With intersecting labour and ecological crises hitting the global food system, there is an urgent need to develop methods to enhance sustainable agricultural production. According to key policy actors, the so-called ‘fourth agricultural revolution’ (‘Agriculture 4.0’), driven by new digital technologies such as AI and robotics, is key to the transformation of the food system to meet the growing global demand for food within planetary limits [1,2].

Public perceptions of new technologies play a critical role in their success or failure [3–6]. Despite an emerging literature exploring the ethical impacts of AI and robotics in agriculture [7–12], there has been little attention paid to public perceptions of these technologies and their implications for the ethical acceptability of AI and robots in agriculture.

This paper draws on a set of twelve dialogue groups conducted across metropolitan and rural Australia to understand how members of the public view the social and ethical impacts of AI and robotics in agriculture. We presented participants with several hypothetical scenarios of AI and robotic technologies – including the possibility of ‘farmerless farms’ – in the horticulture and poultry industries to gauge public attitudes toward these technologies. By mapping these sentiments, we reveal the ethical approaches publics take in assessing the value of agricultural AI and robotics and highlight their importance for research and policy regarding the future of farming.

The paper is organised as follows. First, we briefly introduce the existing literature on the ethics of AI and robotics in agriculture. In the Methods section, we outline our research method and introduce the idea of dialogue groups. We then list the key findings of the study. Specifically, we explore how participant values are expressed in their deliberations about the consequences of the adoption of AI and robotics across the areas of agricultural labour, corporate power, rural identity, and animal welfare. In our discussion, we highlight the significance of our research and point to several areas for further research.

## 2. The ethics of AI in agriculture: Debates and public views

AI and robotic technologies for agriculture range from ‘intelligent’ farm management software, yield prediction algorithms, and smart cameras to machinery such as drones for crop-dusting and monitoring, automated packing and handling systems, and self-driving tractors [11,12]. John Deere now sells a fully autonomous tractor, which can plough a field on a farmer’s behalf and uses AI to collect, stream, and sort images of the areas of its operations. Robotic fruit picking technologies are also in development, with some in trial stages, but are yet to be commercially viable on a large-scale [13,14].

There is a burgeoning scholarship examining the ethical issues raised by AI and robotics in agriculture. Scholars have highlighted issues of data and trust [9,15,16], environmental sustainability [11,17], farmer livelihoods and autonomy [18,19], animal welfare [12,20,21], rurality [22], and labour [23–25].

Our paper builds upon a small body of work that explores public views and perceptions of AI and robotics in agriculture to understand the factors that lead to acceptance or rejection of these technologies [26–29]. It is novel in using the methodology of dialogue groups to generate rich debate among members of the public and in drawing on hypothetical scenarios to induce members of the public to consider the type of agricultural future they value. Our research moves beyond the framing of AI in agriculture as having risks and benefits that create a “trolley problem” [8], and instead draws attention to the meanings and values members of the public ascribe to these technologies when they think about a future in which AI and robotics might be widely adopted in agriculture. Our results add depth and nuance to the existing, mostly quantitative, literature on public attitudes towards agricultural robotics and AI, and constitute a valuable resource for policymakers, or other stakeholders, who want to engage with public opinion regarding these technologies.

## 3. Research methods

The study presented in this paper is drawn from dialogue groups conducted as part of a project examining the social and ethical impacts of AI and robotics in agriculture. Dialogue groups have been used extensively to explore the moral intuitions and ethical evaluations of lay members of the public regarding new technologies [30,31]. Dialogue groups promote facilitated discussions that are centred around hypothetical scenarios in order to elicit participants’ moral positions, and importantly, their reasons for holding such views [31].

To explore public attitudes towards the use of AI and robotics in agriculture, we conducted twelve dialogue groups between August and December 2023. The dialogue group study was approved by the Deakin University Human Research Ethics Committee, approval number HAE-23–068. Participants from metropolitan and regional Australia were recruited by a professional recruitment service (Taverner) using selection criteria based on socio-economic indicators to ensure a diversity of people that represented the communities in the study. Potential participants were then sent an email providing a Participant Information Statement and link to an online Qualtrics consent form to which they provided written consent to participate in the study.

Dialogue groups were held via Zoom and facilitated for 90 minutes. Each dialogue group consisted of four to seven participants. Six dialogue groups involved participants from Metropolitan areas of Melbourne (Victoria), Sydney (New South Wales), and Brisbane (Queensland), with the remaining six groups involving participants from regional farming areas of the Goulbourn Valley (Victoria), New England (New South Wales), and Gladstone (Queensland). All dialogue groups were audio-recorded and professionally transcribed, with participant identities remaining anonymous in the transcription process.

To facilitate discussion, participants were presented with a hypothetical scenario focused on fully automated farms in horticulture and poultry (see S1 File). Scenarios were developed with the aim of provoking discussion of what we took to be key questions about the ethics of AI and robotics based on previous research [11,32], and an extensive literature review. As discussion in each group developed, we introduced complicating factors – such as the shift from semi to fully-automated farms, the removal of farmers from decision-making processes, and biosecurity threats – to tease out why participants held the beliefs they were expressing.

Participants were encouraged to offer explanations for their responses throughout to provide insight into the assumptions, imaginaries, and judgements guiding their positions. We also presented participants with three poll questions at the end of the horticulture and poultry scenarios to produce a quantitative record of their opinions about AI and robotics in agriculture. Dialogue group discussions were audio-recorded and transcribed. Participants were compensated for their time with a $100 e-gift voucher.

We used the Framework Method to analyse the content of the discussions that took place in the dialogue groups [33]. Three researchers from the team read the first three transcripts to develop an initial coding framework. The texts were hand-coded and categorised by deductive (pre-defined codes including the pros and cons of AI, trade-offs, and tensions) and inductive codes. The remainder of the transcripts were then split and coded independently, with an audit being conducted. The process was iterative with discussions held weekly between the three coders and fortnightly with the larger team, with additional themes constructed and added. Codes were charted on an Excel spreadsheet, summarizing the data from each transcript and including references to illustrative quotations [33,34].

## 4. Findings

Dialogue groups were conducted with a total of 67 participants (Table 1). While there was a reasonably good gender split, there were slightly more women than men. Most participants had no direct connection to agriculture although some rural participants had familial connections to agriculture or worked in agricultural and adjacent industries; metropolitan groups were composed of individual consumers. Participants had a range of levels of educational attainment, although, considered as a whole, the average level of educational attainment was higher than that of the average Australian.

**Table 1:** Participant characteristics.

Number of participants = 67	
Age	
18-29	19 (29%)
30-39	11 (16%)
40-49	14 (21%)
50-59	9 (13%)
60-69	10 (15%)
70+	4 (6%)
Gender	
Female	38 (57%)
Male	29 (43%)
Education	
High School	17 (25%)
Trade Certificate	15 (22%)
Undergraduate Degree	22 (33%)
Postgraduate Degree	13 (20%)

This section reports and unpacks participants’ intuitions about the acceptability of AI and robotics in agricultural applications. First, we outline how participants understood the risks and benefits of these technologies. We then explore participants’ ethical positions in relation to four key areas of concern: 1) the future of agricultural labour; 2) rurality and identity; 3) corporate power and; 4) animal welfare and biosecurity. We go on to note the existence of a widespread fatalism amongst participants about the introduction of robotics and AI into agriculture. The section concludes with a presentation of survey results.

### 4.1. Risks and benefits of AI and robotics

Throughout the discussions, participants were prompted to identify and consider various benefits and risks associated with AI and robotics in agriculture, as well as trade-offs between them.

#### 4.1.1. Productivity and efficiency.

Participants typically accepted that AI and robotics would enhance agricultural productivity by overcoming labour shortages. As one participant noted:

“… you also don’t have the issues that you have with workers on site and having to have rest breaks and all that sort of stuff […]. I guess the other thing is it probably increases the income as well because they [robots] get more done perhaps in a shorter time, and it doesn’t cost them as much as it does to actually have people” (Group 4).

Participants also tended to believe that robots were necessary to meet the rising demands for food production in a growing global population, and to boost Australia’s economic and trade position. One participant opined:

“I think it [AI and robotics] gives us a chance to increase the gross volume of what we’re producing, which gives a greater contribution to feed the rest of the world” (Group 5).

However, some participants questioned whether this discourse on productivity may also serve as a ‘trojan horse’ for other agendas. Reflecting on what was driving enthusiasm for AI in agriculture, one participant said:

“Well, if you were going to say to me that if we’re going to get a million tonnes out of a particular area of wheat crop, by going over the AI we can get 2 million tonnes from that same area, then I’d say it’s great. But, if we are just going to get the same quantity of material from the same quantity of land, the only benefit we are getting is to take the labour component out. And I really don’t see the benefit. If it gives a greater product, greater turnover, then I’m all for it. If it’s just a way of cutting back wages, then I think we should have a serious look at it” (Group 5).

Thus, participants tended to be accepting of robotics in the case that productivity is increased. However, there was some uncertainty regarding how likely this is to be realised: such scepticism was associated with the extent to which participants trusted ‘big ag tech’ more generally.

#### 4.1.2. Environmental sustainability.

Some participants saw the precision application of agricultural inputs through AI and robotics as a necessary development, as in the following remarks:

“It probably sounds like a good move in the direction of future sustainability […]. I’m worried that if we stay with our old methods of things, we might run out of food and resources, and I think it’s a change we have to make to continue thriving…, I guess. I’m not worried in this instance, because you still get the fruit at the end of the day, right?” (Group 10).

However, this view was not shared by all. Some participants were concerned about the potential environmental risks and burdens associated with the use of AI and robotics. For example, one remarked:

“First things that I would comment is the environmental impacts of making bigger farms – we’ve seen in America where you can just drive for kilometres and just see one type of tree. And the effect then on pests and diseases that can come in, and you can potentially wipe out a whole orchard… because they’re not broken up, but then also the loss of environmental value for wildlife and insects… and everything else that makes up part of Australia” (Group 3).

The contrast between the views of these two participants suggests that, despite the promotion of these technologies as sustainability measures across the sector, some members of the public remain unconvinced.

#### 4.1.3. Technological reliability.

The extent of participants’ enthusiasm for AI and robotics was also associated with their opinions regarding technological reliability. While there were some participants who saw vulnerabilities as an unavoidable consequence of technological innovation (Group 7), there were others who worried about what they perceived as the fragility of AI and the capacity of robots to work in complex, dirty, and demanding agricultural settings. As one participant put it:

“I was going to say, the downside I see… is machinery breakdown. So, the machines break down, the farmer’s got no labour, crops on the trees waiting to be picked, what are they going to do?” (Group 8).

An overreliance on these technologies is here represented as increasing the vulnerability of farmers, and food systems, to external shocks.

The need for reliable telecommunications and data connections was also identified by some participants as a barrier to the successful application and use of AI technologies. Network outages effecting these technological capacities have recently impacted on farming operations in Australia, garnering media attention in the period prior to the conduct of dialogue groups [35]. One participant reflected:

“If something were to go down, you don’t necessarily have a backup to that. Maybe linked to that somewhat is how reliant the operation… could be on Wi-Fi or other information because that could potentially not always be… at the level that you would need if you’re relying on some sort of – if you were relying on some sort of Wi-Fi, internet data, something. That can be pretty patchy in some parts of Australia as well” (Group 2).

This concern around network stability is arguably a product of the broader digital divide facing rural Australians – and especially farmers.

### 4.2. The future of agricultural labour

Historically, agricultural automation has simultaneously promised improvements to labour conditions and created precarious conditions of employment [24]. Participants were generally positive about the contribution AI and robotics might make to agricultural production by reducing producers’ dependency on unpredictable labour markets. However, many participants also raised a series of ethical concerns related to (un)employment, exploitation, and the transformation of agricultural labour.

#### 4.2.1. Labour shortages and exploitation.

Participants expressed concerns around the effects of robotics upon rural unemployment. As one participant observed:

“It is actually going to affect a lot of people. I know a lot of people that actually do fruit picking as their profession. And, if you are going to introduce the use of AI and drones into picking and into their jobs, it means most of them would have to be laid off” (Group 3).

However, another participant suggested that robots would reduce the amount of time spent “looking for extra workers to try and get the crops picked” (Group 9). Nonetheless, participants did not see productivity benefits as justifying the full displacement of human labour. Instead, these benefits were understood as complementary (Group 3) or working in “parallel” with human labourers (Group 4).

Some participants were also concerned about the broader socio-political and economic implications of robotics and AI, and how it might impact on the conditions of precarity experienced by seasonal labourers working in Australia under the Working Holiday Maker (WHM) visa scheme, and especially Pacific Islanders working under the Pacific Australia Labour Mobility (PALM) scheme. For some participants, there was an ethical question regarding Australia’s responsibility to those displaced by AI and robotics:

“And I think we have some social responsibility, if we’ve been bringing people in from overseas to pick our fruit… maybe we can somehow strategically plan in Australia to address those issues in those Islands…” (Group 3).

Conversely, other participants considered AI and robotics as a means of mitigating the exploitation of seasonal workers. For instance:

“… a lot of the parts of the industry are using modern slavery, that sort of stuff. So, that’s obviously not a good thing. So that [AI and robotics] would work towards hopefully eliminating that” (Group 11).

Another participant suggested that the use of AI and robotics would allow consumers to ‘feel better’ in purchasing produce that was seemingly free from such practices:

“Honestly, when I go shopping and even have a pretty good awareness of agricultural industries, I don’t necessarily think about how that piece of fruit got there. And then, when I think, oh that’s bad, I don’t think about those poor people who pick fruit who might be being exploited by the labour hire company. I actually feel better about it being picked by a robot, perhaps because I’m not sure that the fruit-picking industry at the moment might not necessarily have the best employment” (Group 2).

These differing positions illustrate how participants mobilised arguments about the relation between robots and AI and vulnerable seasonal workers to reach different conclusions.

#### 4.2.2. Social value of agricultural labour.

When discussing agricultural labour, participants also ascribed different values to different types of workers and jobs, which then influenced how they viewed the impacts of AI and robotics. On the one hand, these technologies were seen as offering *poultry workers* a way out. As one participant noted:

“… there’s a bit of yuck about working in a chicken farm, too. Most of us wouldn’t really want to do that as a career choice from the start, so yeah, there are things that are definitely yuck about that. It must not always be a pleasant environment for human beings, so it’s much better to have machines doing it” (Group 1).

Another participant represented the risk of technological unemployment in the poultry sector as an opportunity “to find more interesting work for the people that are in those situations, rather than use them as pack mules” (Group 5).

One the other hand, many participants saw AI and robotics as offering a pathway for skill development and new employment opportunities in the *horticultural* sector. One participant considered that automation would “actually make the job more enticing” for younger generations with links to agriculture but trained in computer science (Group 6). Automation would then expand what skills are necessary to work in agriculture.

### 4.3. Rurality and identity

The potential of AI and robotics to transform agricultural roles also led participants to draw and reflect upon what it meant to be a farmer and about the character and future of rural communities.

#### 4.3.1. Who is the farmer?

For some, the possibility that farmers might become more like ‘technicians’ was not viewed as a loss, but rather as a natural progression, as per the following remark:

“I actually found it interesting, because I don’t particularly agree with it in the sense that I don’t see it as we are losing farmers, but it’s more that farmers will still be a part of the process” (Group 7).

Promoting the emerging role of ‘farmer-technician’ was also seen as a way attract more Australians to jobs in agriculture. As one participant shared:

“For some reason in Australia, the mindset is that Australians don’t do that type of work, the picking and that kind of thing. That’s something that I’ve noticed since I’ve moved here. But I think that Australians would do technician jobs in that area, technical things. So, maybe it’s making Australian workers more employable in that field” (Group 1).

Nonetheless, there were many participants who continued to valorise a more traditional vision of what it is to be a farmer. For these individuals, the shift towards fully automated agriculture was viewed as a significant social and cultural loss:

“A human farmer who depends on a farm to live off their life, that’s their generation – that’s their family business – is now taken away and you’re now a technician. It sounds a bit dystopian to me… and I just think that’s too far” (Group 6).

Another participant echoed this sentiment, suggesting that the “pride of being a farmer” would be lost through full-automation, and farmers would then become “just like workers on their own land” (Group 11).

This latter set of participants often seemed to see farmers as stewards of the land, who have an intuitive knowledge of local conditions that could not be achieved by a technician that controlled farm operations remotely. This responsibility of farmers is clearly expressed in the following remark:

“… we’re farmers, we care about the land in a way that a technician in LA [Los Angeles] could hardly remotely do […]. And although people think we exploit the land, of course we don’t. We’re the ones that are really aware of the other animals and plants that share the land that we’ve got” (Group 11).

#### 4.3.2. Agrarian identity and human purpose.

In countries like Australia, where the idea of the farmer has been central in constructing an origin story and national identity [36,37], AI and robotics complicate rural imaginaries more generally.

In one group, participants discussed how the shift towards more automated forms of agricultural labour would subsequently impact on an important “way of living” (Group 3), with one participant drawing on:

“… the old image of the farm… the pig in the puddle of mud and so on, that’s a, it’s a very human romantic thing that’s in the past, but it’s part of what makes us human” (Group 5).

While this participant was aware of the nostalgia driving this imaginary, what seemed to be more important was holding onto it as a way to find meaning in human life. This was reiterated, as another participant questioned: “what kind of world will we live in if it’s all automated?” (Group 2).

Several exchanges also revealed that many participants believed that it was important to maintain connections to the natural world. Despite – or perhaps even because of – many participants being divorced from direct farming practices, their reservations about AI and robotics often stemmed from the belief that we should know where our food comes from to avoid “totally distancing ourselves from that side of things” (Group 5). One participant tackled this tension specifically in the context of poultry, suggesting that:

“… I mean it’s gruesome. But having eggs go in one side, and then chicken meat come out the other, and no humans involved, to me that’s maybe a little bit too – we’re disassociating ourselves just a little bit too much I think from where the stuff comes from” (Group 6).

As another participant insisted that “to work in a chicken farm, you have to find out that, yes, this is a living thing that’s going to be killed for you to eat” (Group 5).

#### 4.3.3. Rural collapse and agricultural nostalgia.

An idealisation of rural life also played a role in shaping participants’ thinking about the effects of AI and robotics on farming communities. Many participants worried about how rural townships would change as a result of AI and robotics putting local people out of work, with one participant arguing:

“To me, it would be like hollowing out the country part of Australia. And what would our towns look like? Where would people go if they no longer had something to keep them anchored in the towns” (Group 1).

Similarly, another participant reflected:

“If there’s fewer people in the community earning a living then the flow down the line doesn’t remain. So, it could be – I don’t think it will be the end of towns, but of course, if there’s fewer and fewer job opportunities, people will start to leave again” (Group 11).

### 4.4. Corporate power

As agri-tech continues to advance and develop new capacities through AI and robotics, there are new convergences of ‘Big Tech’ and ‘Big Ag’ that further amplify questions about corporate power over the food system [11,38–41]. Throughout the dialogues, participants demonstrated an awareness of these questions.

#### 4.4.1. Concentration and monopolisation.

Strikingly, some participants viewed AI and robotics as a “Pandora’s Box” that would bring with it a host of unintended consequences around the corporate structures of agriculture (Group 11). As one participant observed, “once the technology is out there and people start adopting it, if competitors don’t do the same, then they’re just going to suffer anyway” (Group 11).

Other participants associated this ‘suffering’ with the costs associated with adopting AI and robotics:

“… farms that can’t afford that sort of technology, or that are run completely by humans, if they’re completely lagging behind does that then eradicate their business… or does it make it really hard to be competitive because maybe costs are different?” (Group 4).

For some participants, then, AI and robotics were seen as a potential tool to price out smaller-scale farmers, and therefore, further entrench corporate control of agriculture. One participant reflected:

“… my biggest fear I guess… is just monopolisation. To me, it’d just be one company would end up owning all of the farms, and it’d just become one big monopoly, and there’d be no competition, and they’d be able to charge what they want” (Group 9).

Moreover, in the context of discussions of corporate power, some participants were sceptical as to whether the supposed benefits to consumers from robotics and AI, in the form of cheaper food would actually be realised. One participant remarked of this prospect:

“… in an ideal world that would be fantastic… if those companies actually thought that way. But unfortunately, they’re profit-driven, they think about their shareholders – if they have shareholders – or their bottom line. They don’t really think about the community cost in the area that they’re making money” (Group 5).

Such scepticism led one participant to explicitly reject the introduction of these technologies into agriculture “so that the top 5% can make more profit” (Group 2).

#### 4.4.2. Ownership and control.

Participants expressed specific concerns about the ownership, control, and autonomy of farm operations. In our hypothetical scenario of farmerless farming in horticulture, we raised the possibility that farming decisions might be made by operators overseas – a prospect about which many participants voiced their apprehension. One participant went so far to say that they thought that these types of decision-making structures were “fraught with danger” (Group 1).

Anxiety around control of AI-enabled agriculture was accompanied by concerns about foreign ownership of agricultural land. As one participant reflected on the risks of AI in agriculture:

“… one of the downsides would be if it’s farmland given to foreigners. So, I would say I would prefer to get produce where the vegetables are grown by Australians on Australian land” (Group 11)

These concerns about ownership were also raised about the ownership of AI and robotic equipment, as stated by another participant:

“… once you’ve outsourced your labour to a company that provides you with machines to do your labour, and you don’t own those machines, then you’re beholden to them and whatever they charge […] – so the autonomy and agency, in terms of running your business, is removed from that” (Group 11).

Participants also raised several questions regarding who becomes responsible and accountable for AI and the associated data. As one participant observed:

“… in today’s economy we’ve had a lot of leaks of late with just basic data. But this would be more intense data, and this would be affecting our livelihoods in a sense that this is potentially all about agriculture. So, I think that’s quite scary to know, who would have their hands on that data? How can it be shared? What can be done with that information?” (Group 7).

These concerns around data leaks expanded into the domain of national security. Although considering it a “more apocalyptic doomsday thing”, one participant feared that all it would take was for the “big bad” nation of the day “to create flaws in the algorithms” and then hold our agricultural production at “siege” (Group 5).

### 4.5. Animals, AI, and robots

Participants also attempted to navigate the interests of human and nonhuman animals as effected by full automation in poultry. In their deliberations about the prospect of full automation of poultry farming, participants struggled to balance the human benefits of improved biosecurity and its effects on the welfare of poultry.

#### 4.5.1. Animal welfare and the free-range problem.

Participants were broadly accepting of the idea of full automation of poultry production, due partly to their belief that much of the industry had already been automated. However, their acceptance was contingent on animal welfare being considered in the application and use of AI and robotics. One participant insisted:

“… the number one thing, even before price, would be animal welfare. And if they could prove that the animals were looked after properly to the best possible standard, that would be fine… to me, the number one thing is the animal welfare and proper certification” (Group 8).

The importance of animal welfare was mobilized in arguments both for *and* against automation. For instance, some participants argued that the current industrial system was already “robotic” (Group 9) and that the removal of humans might actually improve the welfare of birds in poultry farms by preventing human cruelty, as one participant shared:

“There’s good and bad people out there. And I’m guessing the robots would be very consistent… the chickens might grow being more relaxed, and maybe even produce better quality meat and get sick less because of better treatment” (Group 10).

However, others were less sanguine, as per the following remarks:

“If they were fully automated what would happen to the current circumstances where we have the free-range farms and the battery farms where the chickens are in the cages forced to lay, or you’ve got the free-range chickens that were allowed to roam? What would happen with that? Because the way that I would foresee it is that it would become less humane and become more that everything would be battery farming, so to speak” (Group 7).

Other participants worried about how the use of AI and robotics in poultry farms might devalue animal life. Considering a scenario whereby fully automated poultry farming would require housing poultry indoors, one participant questioned:

“… if they’re not in their natural environment, what impact does that have on the animals? And what does that say about us as a society? Does it say that we care more about the profits or whatever that we can generate from these animals, than we do from their welfare?... I just find it a bit disturbing” (Group 4).

Accordingly, some participants emphasized the importance of alternative forms of production that emphasised “human interaction with our animals” in small-scale enterprises, which, they argued “has actually led to better production, better reproduction on farms, [and] egg production increasing” (Group 3).

#### 4.5.2. Biosecurity.

While in other contexts, participants were uncomfortable with the idea of removing human beings from farms, many participants accepted that complete automation was the best way to address biosecurity concerns in poultry production. As one participant noted of the current industrial poultry sector: “more chickens, more people, more possibility of problems just in numerical terms” (Group 8), with another suggesting that the poultry sector would see “positive benefits for humans by removing humans” (Group 1).

However, some participants pushed back against this suggestion:

“If you’re trying to jam more chickens into the same sized areas, well, you shouldn’t. You’re going to up the chances that something’s biologically going to go wrong at some stage, aren’t you?” (Group 2).

Similarly, another participant commented:

“I would say, based on the story that I’ve heard so far, having AI is reducing the amount of space that poultry farming might take by cramming them in a little bit closer, which increases the spread of diseases. And it’s also like playing God in essence, but in a bad way because evolution happens and it’s a breeding ground for all these diseases that might grow” (Group 11).

Like animal welfare, then, the importance of biosecurity was used to mount arguments both for *and* against automation.

### 4.6. Scepticism about public involvement in shaping the future of agriculture

Despite these various concerns, participants were overwhelmingly fatalistic when it came to the future trajectory of the food system. This trend was viewed as being driven by the agricultural and tech companies, rather than by consumers or citizens, as expressed by one participant:

“… the next time you go to the supermarket, there’s a board saying, ‘robot picked fruit’ and ‘robot picked eggs’ or whatever. And you’re paying 10 cents extra for the human picked ones or whatever” (Group 8).

Some participants saw automation as something “already happening” (Group 4), or in the process of becoming the norm for agriculture (Group 5). Several participants described changes in their communities that they identified as being caused by the encroachment of automation and farm consolidation; for instance, the demographic makeup of rural towns had shifted to retirees and “weekenders” rather than people working on farms or in agricultural service industries.

### 4.7. Survey findings

Participants were presented with a series of survey questions at the conclusion of discussions (Table 2). The only items that secured more than 50% support were the use of AI in horticulture justified as a business measure and as a sustainability measure and in poultry production for the sake of biosecurity. Interestingly, despite just having spent time discussing each example, a sizeable number of participants remained neutral about the appropriateness of using AI and robotics for profitability, biosecurity, and animal welfare when they were required to state a final position in their response to the questions in the poll.

**Table 2:** Poll results.

To what extent do you agree it is appropriate to prioritise bigger farms with fully automated farming to increase profitability, reduce resource use and produce cheaper food?	
Agree	28 (43%)
Neutral	17 (26%)
Disagree	20 (31%)
To what extent do you agree that fully automated horticulture is justified as a business measure?	
Agree	34 (52%)
Neutral	14 (22%)
Disagree	17 (26%)
To what extent do you agree that fully automated horticulture is justified as a sustainability measure?	
Agree	42 (64%)
Neutral	9 (14%)
Disagree	14 (22%)
To what extent do you agree it is appropriate to prioritise fully automated poultry farming to increase profitability, reduce labour costs and lower consumer cost of poultry-meat and eggs?	
Agree	31 (47%)
Neutral	13 (20%)
Disagree	22 (33%)
To what extent do you agree that fully automated poultry production is justified as a biosecurity measure?	
Agree	45 (68%)
Neutral	13 (20%)
Disagree	8 (12%)
To what extent do you agree that fully automated poultry production is justified in terms of animal welfare?	
Agree	22 (33%)
Neutral	23 (35%)
Disagree	21 (32%)

## 5. Discussion

Society has always contained those who have “praised” and “glorified” new technologies and those who are critical of new technologies and their associated social, ecological, and political impacts [7]. The participants in the dialogue groups convened for this study did both of these things.

One thing our study shows, then, is how deep and pervasive disagreement about the ethics of the application of robotics and AI and agriculture is. Agricultural robots and AI are almost always portrayed in a positive light in both popular and academic contexts [42,43]. A recent study [29], conducted in Germany, suggested that the public is generally positively inclined towards agricultural robots, despite the long history of scepticism towards new technologies in Germany on environmental grounds. In contrast, our study suggests that a significant portion of participants were deeply sceptical of the use of robots and AI in agriculture. Moreover, the extensive, and often enthusiastic, discussion between participants that took place in the groups did not alter this or produce consensus. As the poll results showed, somewhere between (roughly) a quarter and a third of participants disagreed with the full automation of agriculture in horticulture and/or poultry production for any reason other than to improve biosecurity. A similar, if slightly smaller, proportion remained neutral on each topic. Our results therefore suggest that, at least in Australia, public opinion is more divided than other studies have suggested.

Another significant finding was that different participants often mobilized the same values in support of arguments both for *and* against the use of robotics and AI in agriculture. That is, even when they agreed on the importance of a particular value – for instance, environmental sustainability or animal welfare – participants often disagreed about whether it constituted a reason to be enthusiastic about the adoption of robotics and AI in agriculture, or a reason to try to be opposed to their adoption. Appeals to the same value in service of arguments to different conclusions occurred in the context of discussions of the environmental impacts of robots and AI, of their implications for labour, and the contribution robots and AI might make to biosecurity. Some participants held that robots and AI would reduce the environmental impacts of farming by facilitating precision watering and application of fertilisers, while others thought that they would threaten biodiversity by promoting monoculture. Some held that robots and AI will free farmers from having to rely on an increasingly scarce pool of seasonal workers, while others thought they would generate technological unemployment in rural areas. According to some participants, these technologies will be good for workers by relieving them of the need to do “dirty” jobs and creating a need for employees with technical skills; according to others, they will bad for workers by rendering them more vulnerable to surveillance and exploitation [44,45]. Further, participants believed *both* that robots might improve the welfare of poultry by reducing their vulnerability to cruelty from human workers and eliminating interactions with people that are stressful for birds *and* that they might threaten the welfare of poultry by confining them *en masse* in highly artificial environments: each of these claims finds some support in the growing body of literature exploring the implications of AI for the interests of nonhuman animals [46–48]. Even the value of biosecurity, which appeared as the motivation for a hypothetical full automation of poultry production in one of the scenarios that the participants in the dialogue groups were asked to consider [12], was mobilised by some participants as a reason *not* to proceed down this path.

Thus, rather than serving to motivate consensus, these shared values served as sites for disagreement about the merits of different ethical and technological futures. These observations suggest that it will be difficult for either advocates *or* critics of automation in agriculture to win the public over by framing the issue in terms of the importance of any particular ‘trump’ value. They also suggest that much sociological and philosophical work remains to be done in identifying, untangling, and evaluating different arguments that appeal to each of these different values.

Interestingly, one attitude – if not quite a value – which did unite (most) participants was scepticism about the real corporate agenda behind the push for the use of robots and AI in agriculture. A surprising number of our participants might be characterised as what Langer and Kühl identified as “skeptical citizens” [26]. That is, they were sensitive to the risks posed by the introduction of AI and robotics – particularly when it came to its implications for farmers on small farms – and inclined to question the contribution technology more generally was making to human progress. Participants expressed this scepticism by means of cynical remarks about corporate agendas, and by questioning whether the goods that were being promoted as being served by the introduction of robots and AI were really what was motivating those developing and advocating for these technologies. It has been suggested that many of the digital technologies being touted as ‘solutions’ to the problems facing agriculture are being developed by Silicon Valley entrepreneurs with little understanding of the sector, and are often rebranding versions of products developed for other purposes [49]. Even when agri-tech startups set out to develop products with the goal of promoting sustainability or social goods, commercial imperatives often come to dominate the final version, especially if the startup is bought out by a more established market player (for an example and useful account of this dynamic, see [50]. The confluence of ‘big Ag’ and ‘big data’ produces new corporate conditions that risk exacerbating the uneven flow of benefits throughout the food system, further disenfranchising farmers and strengthening multinational corporations [9,38,51]. Thus, the serious concerns expressed in the dialogue groups about the possibility that these technologies might lead to further consolidation of ownership of agricultural enterprises and disempower farmers in relation to decisions about how they farm, receive some support from the literature. The discussions that occurred in our dialogue groups suggest that, in Australia at least, manufacturers of agricultural robots and AI suffer from a significant trust deficit in the eyes of the public.

Our results also foregrounded questions regarding how, and when, the interests of certain groups become centred in the ethical evaluation of AI and robotics, and how and when others are rendered marginal. It was notable, for instance, how participants referred to the interests of Pasifika workers in discussions about the implications of the adoption of robotics and AI for the exploitation of labour, but to the interests of Australian citizens when it came to talking about the potential of these technologies to create opportunities for skilled work in rural areas. There are genuine concerns regarding the conditions of employment of Pasifika workers in the Australian horticultural industry which are usually precarious and sometimes verge on a modern form of slavery [52,53]. However, the distinction in types of labour – that is, the skilled ‘farmer’, and unskilled and exploited ‘migrant worker – that was mobilised by some participants plays into the ongoing racialisation and ‘production of precariousness’ that shapes the experiences of, and the opportunities available to, Pasifika and other migrant agricultural workers [54,55]. Our findings therefore highlight the importance of interrogating not only whose interests are considered in the contexts of debates about any transition to AI and robotic agriculture, but also how these interests are constructed and to what ends. They also speak in favour of including immigrant farm workers, whose experience and working lives will be reshaped by technological change and who are often “invisibilised” in this context [56], in deliberations about the future of farming.

Participants also drew on longstanding nationalist narratives about the role played by agriculture in securing Australia from the “threat” posed by foreign powers and values. This was, perhaps, unsurprising given that concerns about foreign ownership of Australian land and financial investment in agriculture in Australia have received considerable media attention in Australia over the last decade [57–60]. Nevertheless, appeals to the importance of maintaining national sovereignty had considerable rhetorical force in the context of participants’ discussions of the implications of adopting AI and robotics for control over agricultural land, technology, and data. These appeals sat uncomfortably next to calls for Australian farmers to make the transition to a more automated agriculture in order to maintain their global position in providing reliable, safe, and nutritious foods to a growing global population.

Relatedly, it was clear that participants were often influenced by a powerful and nostalgic imaginary regarding the nature of rural life and the role of the farmer in discussions. In Australia, agriculture and farming – and thus, the ‘bush’ – plays a significant role in the shaping of a national identity that is grounded on the valorisation of the (settler) farmer as a hardworking, steadfast, and resilient embodiment of stewardship and good citizenship [61]. This social imaginary played a significant role in shaping discussions, with a number of participants suggesting that the introduction of robotics and AI into agriculture would impact on the nature and quality of rural life and increase the vulnerability of rural towns to external economic, political, and social pressures. Implicitly – and sometimes explicitly – life in rural areas was figured as different from, and superior to, life in big cities: this ‘idyllic’ rural way of life was what needed to be defended from the impacts of new technologies [62]. Despite many of the participants having no direct experience of farming, they mobilized a figure of the farmer as someone with a distinct social role, a historical tie to a particular area or piece of land, and a strong moral character in order to think about the future of farming [37,63]. Again, this idea – this imaginary – of the farmer often functioned as something that needed to be preserved and protected against the encroachment of a new, more technical, more technological vision of food production. Whether this idea of the farmer and farming is realistic and/or worthy of preservation strikes the authors as being and important ethical and political question when it comes to the larger debate about the future of farming.

Insofar as food systems affect and implicate all of us, the case for public, democratic, input into the future of the food system, including decisions about the technologies that are likely to shape it in the future, is especially strong [64]. Calls for public deliberation, citizen engagement in science, and democratic input into technological trajectories are rife in the Responsible Research Innovation and Science and Technology Studies literature, including the literature on agricultural robotics and AI [32,65,66]. Several of our findings – that the public is divided in its attitudes towards the use of AI and robotics in agriculture; that this difference persists even when those with opposing opinions appeal to the same values; the diversity of different ethical and political concerns expressed by participants; and that members of the public are sceptical about the motives of those developing these technologies – lend force to the idea that there is a strong moral, as well as a pragmatic and political, case for including the public in decision-making about the future of agricultural robots and AI. The detail of our findings, especially regarding the arguments that participants put forward in the discussions, constitute a valuable resource for policymakers or other stakeholders who want to engage with public opinion on these technologies.

In this context, it is, perhaps, unfortunate that our participants seemed to have little faith that consumers and citizens would have any say regarding the future shape of food systems. Despite their seeming enthusiasm for discussing the ethical issues of AI and robotics in agriculture, participants seemed resigned that the sector was already heading in the direction of fully automated farming. Given the strength of the case for public input into the future of food systems, this struck the authors as both depressing and disappointing.

Inevitably, this study has some limitations. Insofar as the dialogue groups method relies on the introduction of various scenarios by the research team, concerns about ‘framing’ have some valence here [67]. Given that participants did not necessarily know much about the current state of AI and robotics in agriculture when commencing discussions, there was some risk that their attitudes were significantly shaped by the scenarios we presented, which postulated capacities of robots and AI that have not yet been seen at commercial scales. Regardless of the role – if any – played by the scenarios in shaping participants’ responses, the fact that most people have little experience with robotics and AI means that their ideas about these technologies tend to be shaped by their representation in science fiction [68]. This is an unavoidable limit of any contemporary research regarding public attitudes towards AI and robotics. The authors believe this risk was justified by virtue of the method revealing participants underlying moral and political values, which they brought to a discussion of scenarios that were explicitly flagged as hypothetical.

Similarly, it is possible that the poll results exaggerate the extent of public hostility to the use of robots and AI in agriculture by virtue of asking participants about their attitudes towards “full automation” in the context of horticulture and poultry production. We did this in hope of eliciting strong responses to the prospect of increased use of robots and AI in agriculture, which might in turn move participants to reveal the values they brought to the question. Wording the survey questions differently, for instance to ask participants about their attitudes towards an “increased” use of AI and robotics, might have generated a different set of responses.

As we observed above, a significant number (between 13% and 35%, depending on the question) of participants chose to record their attitude towards the use of robots and AI in various forms of agriculture as neutral. A limitation of the survey instrument is that, by itself, it does not allow us to distinguish between participants who might have been undecided on, or felt insufficiently informed about, the use of these technologies, and those who might have reached a confident conclusion that the considerations speaking for and against the use of these technologies in agriculture were precisely balanced. That said, the dialogue group methodology used in our study facilitated wide-ranging discussions about the merits of the use of AI and robotics in agriculture, which provided ample opportunities for participants to ask questions of each other and/or the facilitators. We believe it would, therefore, be a mistake to assume that those who chose to record their attitudes as neutral were not reporting a considered conclusion.

Obviously, there is also a question as to how much our findings, based on research conducted in Australia, generalise to public attitudes towards the use of robotics and AI in agriculture in other nations. Given the similarities between the economic and environmental challenges facing farmers in Australia and in other nations that practice forms of agriculture that are amenable to automation, we suspect that our findings may well be relevant to discussions of the ethics of the use of robotics and AI in agriculture in other nations as well: this awaits confirmation by the results of the work of others.

Finally, while we have attempted to attend to some of the ethical and political issues arising out of the history of race in the context of agriculture in Australia, we did not explicitly refer to Indigenous perspectives in our scenarios. Further research, on the intersections between agriculture, race and coloniality, and emerging technologies, will be necessary to explore the implications of the history of invasion in Australia, and the attitudes of contemporary First Nations people, for the future of agriculture.

## 6. Conclusion

Despite media and industry actors celebrating the promise of AI and robotics for agriculture, our study finds that many members of the public remain sceptical of these technologies. Participants were concerned about how the food system ought to be structured and drew on longstanding agrarian imaginaries in defence of the moral value of farming: racial and nationalistic determinants of this imaginary also influenced how and when participants considered the interest of various groups who might benefit from, or be harmed by, the use of robotics and AI in agriculture. Although participants could agree on the values that should be brought to the discussion about the future of agriculture, they disagreed on whether these values spoke in favour of, or against, automation. When required to express opinions about the prospect of farmerless farms, our participants were divided, even after lengthy discussions.

By revealing the extent of differences in opinions regarding the wisdom of adopting AI and robotics in agriculture, our research provides support for the idea that there is a significant question concerning the democratic legitimacy of policies regarding the development and use of these technologies. If – as we believe is appropriate – policymakers and technology developers want to ensure that future applications of robotics and AI in agriculture have social license, then they must engage with, and take seriously, the range of public opinions on this topic. In reporting the diverse concerns expressed by participants in their own words, our study provides a valuable resource to policymakers, or other stakeholders, who wish to do so.

## Supporting information

S1 File Dialogue Groups Scenarios (attached file). (DOCX)
