# Supplementary material for: “You’re opening Pandora’s Box”: Public attitudes on AI and robotics in Australian agriculture
Source: PLoS One. 2025 Sep 15;20(9):e0332461. doi: 10.1371/journal.pone.0332461 (PMC12435636; doi:10.1371/journal.pone.0332461)
Supplement: S1 File — (DOCX) [file pone.0332461.s001.docx]

**Supplementary Information – Dialogue Group Scenarios**

**“You’re opening Pandora’s Box”: Public attitudes on AI and robotics in Australian agriculture**

*Agriculture and Human Values*

**SCENARIOS FOR DIAGLOUE GROUPS – fully automated poultry farms**

**Background Information**

Australian agriculture is currently facing profound challenges because of soil degradation, water scarcity, loss of biodiversity, climate change, changes in the composition of rural communities, and threats to biosecurity.

At the same time, researchers are looking at using robots and artificial intelligence (AI) to improve agricultural production.

AI and robotics promise to make farming more efficient by determining the precise amount of water and fertilizer needed in each paddock, robots can carry out labour intensive tasks like weeding, fruit and vegetable picking, food handling, packaging and so on.

Therefore, AI and robotics offer significant benefits to the agricultural sector.

Even though there has been considerable research into the benefits of using these technologies, not much attention has been given to the social and ethical impacts that this shift in agricultural production could generate.

There are many proposed uses for AI and agriculture, one is fully automated farms.

**So now I am going to talk through some scenarios about poultry farms and Orchards or fruit farms.**

**Let’s start with fruit and vegetable farms broadly know as horticulture.**

**Scenario 1**

Australia's horticulture industry comprising fruit, vegetables and nuts, is one of the nation's largest agricultural industries. Horticulture makes a $8.6 billion dollar contribution to Australia’s economy.

Fruit and vegetables are essential to a healthy diet and the horticultural industry makes an essential contribution to the health and well-being of the Australian community.

Many fruit and vegetable farms remain relatively small. However, the size of farms is increasing due to economies of scale associated with new technologies.

The industry is very competitive, both in Australia and overseas. It also has labour and seasonal challenges – especially with the impacts of climate change and climate variability.

Labour shortages can challenge the profitability, economic contribution, and sustainability of agriculture industries. The Australian Seasonal Worker Programme was set up in 2012 in order to contribute to the supply of low-skilled labour from the Pacific Islands to Australian horticulture industries.

The money these seasonal workers send back to their home countries are a valuable form of household income. They enable improvements in the lives of workers and their families by providing money for school fees, housing improvements, and community projects in their home countries.

The Australian towns around the farms that employ seasonal workers also benefit from the multiplier effect of spending by seasonal workers on goods and services – also building positive relationships between season workers and local communities.

However the availability of seasonal labour is becoming much more inconsistent which has major implications for the viability of horticulture as a business.

On the flip side of this are repeated cases of systematic and sustained worker exploitation – where seasonal workers and backpackers have effectively been paid as little as $2.50 an hour, with some labour hire companies holding people’s passports in circumstances that amount to bonded slavery.

The future looks complicated. Competition is increasing due to imported produce, variability in produce prices and on top of all this climate conditions are also changing.

To stay successful, the industry is experimenting with new production methods and increasing the scale of production.

**Among these innovations is the development of fruit picking robots - like this prototype designed in Australia** [**https://www.youtube.com/watch?v=IzaaSIEDg7s**](https://www.youtube.com/watch?v=IzaaSIEDg7s)

The model of supply that robot developers are working on is the same as that used by seasonal labour hire companies – the robots will be owned and operated by a fruit picking company who will be paid by the bin of produce picked. This system of payment is the same as many of the season worker hire companies currently operating.

For farmers using companies that provide robots that can pick fruit and vegetables offer the possibility of:

- More profitable horticultural industry
- Avoiding delays to harvesting associated with the difficulty of securing seasonal labor.
- Reduced labor costs
- Cheaper fruit and vegetables for consumers

It is, we believe, time to think about whether this project should be pursued.

**Just pause for a minute and think about a scenario in which most crops are harvested by robots**

- Is the prospect of most fruit and vegetables being picked by robots appealing? Are there good / bad things about it?
- If you worked on a fruit or vegetable farm, what concerns would you have about the introduction of automated systems?
- As a consumer would you have any concerns?
- Are there other factors or things you think need to be considered?

**Scenario 2**

So now let’s expand the horticulture story a bit.

Ownership of agricultural land and capital has become increasingly concentrated in Australia over the last several decades owing to economies of scale associated with the use of new technologies.

The introduction of robots is likely to accelerate this dynamic. Robots work best when the task can be changed to suit them. This may mean bigger farms, more crops grown in greenhouses, or replanting orchards to suit robot picking.

The other technology that could improve productivity and farm viability is artificial intelligence and precision agriculture.

In greenhouses, temperature, ventilation, and lighting can all be controlled remotely, or even automatically, and adjusted for maximum productivity and minimum resource wastage – so more food is produced per hectare using less water and fertilizer.

These types of systems can potentially be scaled up. Data from drones, soil sensors and other surveillance technologies on an entire farm could be processed by computers that will then make decisions about how best to maintain the health and productivity of trees/bushes and the ripeness of fruit or vegetables. “Precision watering” systems deliver calculated amounts of water and fertilizer to particular areas or particular plants.

Eventually, these types of systems would remove the need for local farmers to make ‘farming’ decisions – this could be done from anywhere in the world.

For example some-one in an office in Los Angeles or Singapore could be operating a farm remotely in Australia

If we take it another step again - these types of systems could eventually remove the need for humans to make any ‘farming’ decisions – the Artificial Intelligence would direct agricultural activities on the farm based on the real-time data being collected and fed to it with no human involvement.

The people who worked on farms would not be farmers but technicians who’s only role is to maintain and service machines, sensors and other technical systems.

**Just pause for a minute and think about a scenario in which farming is all done by machines**

- Is the prospect of large farmerless farms appealing? Are there good / bad things about it?
- If you worked on a fruit or vegetable farm, what concerns would you have about the introduction of automated systems?
- What would completely automating horticulture do to rural communities?
  - Good things / bad things
- As a consumer would you have any concerns?
- Does it matter if decisions about what to farm and how to farm it are made in another country?
- Does it matter that we no longer have farmers – something human societies have relied upon for the last 10,000 years?
- Are there other factors or things you think need to be considered?

***RUN POLL 1***

**Ok thanks for your thoughts on this – now we are going to reset and look at poultry farming**

**Scenario 3:**

Poultry production makes a substantial contribution to global food security, providing energy, protein, and essential micro-nutrients to humans.

The poultry sector is expected to continue to grow as demand for meat and eggs is driven by growing populations, rising incomes and urbanization.

Increases in productivity in poultry farming have been achieved by intensification:

- an expansion of the scale of production
- and the development of more productive breeds of chickens.

Modern poultry farms are big. Intensive meat-chicken farms may house several hundred thousand meat chickens at any one time.

Farms producing eggs may be larger still, with some facilities in China now containing millions of layers. Turkeys, ducks, and geese are also increasingly farmed in intensive production facilities.

Another way of increasing productivity and efficiency of poultry farms is the use of automation. Many poultry facilities are already highly automated. Eggs may be candled, sorted, and vaccinated by a robotic system and hatched in automated incubators.

Temperature, ventilation, and lighting can all be controlled remotely, or even automatically, and adjusted for maximum productivity.

Food and water are provided using automated systems that deliver precise amounts of each and so on …

However, there remains several tasks essential to poultry farming that still require that people enter the facility. Further innovations in robotics and automation are required before it might be possible to introduce robots and automated systems that eliminate the need for human beings to be involved in poultry farming completely. But these are in development.

Given the rate at which robotics and AI are developing in the current period, the prospect of farms on which human beings never set foot no longer seems far-fetched.

Fully automated poultry farms offer the possibility of:

- More profitable poultry industry
- Less stressful / better environments for chickens more generally
- Reduced labor costs
- Cheaper chicken-meat and eggs for consumers

**Just pause for a minute and think about that scenario**

- Is the prospect of complete automation in poultry farming appealing? Are there good / bad things about it?
- If you worked in a poultry farm, what concerns would you have about the introduction of automated systems?
- As a consumer would you have any concerns?
- Are there other factors or things you think need to be considered?

**Scenario 4:**

Ok so let’s change the chicken farming story a bit.

Large scale poultry production also provide ideal conditions for the rapid evolution of bacteria and viruses.

In particular, they are sites which can generate highly virulent strains of Avian Influenza or Brid Flu. Outbreaks of these highly virulent strains of bird flu in commercial poultry production disrupt international trade, decrease income in nearby communities, interrupt farm operations, and cost government and industry huge amounts of money to control.

For example, the US governments spent more than $879 million dollars to control the 2014–2015 Bird flu epidemic, making it the most costly animal health incident in US history. Fifteen years after its emergence, the direct economic costs of this bird flu outbreak – including destroying more than 250 million birds – were estimated by the World Bank at more than US$10 billion

Evidence suggests that the persistence of bird flu in poultry flocks is a function of farm size, and that current production systems are increasingly configured in ways that can sustain the circulation of this virus indefinitely.

Poultry farmers have three strategies to try to control bird flu.

1. They try to mitigate outbreaks by slaughtering infected birds, cleaning and disinfecting poultry houses, and minimising movements of birds or staff between different areas in the same farm or between farms
2. They can vaccinate birds. However, the vaccines are not that effective, are expensive, and are not a substitute for good biosecurity. Vaccination can also drive the development of new more virulent strains of bird flu.
3. Farmers can also try to prevent bird flu entering the facility by removing points of contact between poultry flocks, wild birds, and potential human vectors. Preventing contact with wild birds and their droppings requires that poultry be confined in cages or to barns.

As well as killing birds, Bird flu can infect humans causing human deaths and illness.

For instance, over the last 20 years Avian influenza strains caught from poultry have caused the deaths of more than a thousand people.

While, as yet, these strains of bird flu are not easily transmissible between human beings, the possibility that a highly transmissible variant might emerge is extremely concerning.

Fully automated poultry production would allow farmers to prevent bird flu entering the facility by preventing any interaction between poultry and other birds, humans, or other vectors.

Removing all contact between human beings or wild birds and domestic poultry is also potentially a critical step in preventing an influenza pandemic in the human population.

**Just pause for a minute and think about that scenario – given the risks to food production and human health posed by bird flu …**

- If you worked in a poultry farm, what concerns would you have about the introduction of automated systems that completely removed all contact between chickens, wild birds, and humans?
- If you owned a poultry farm would the prospect of complete automation be appealing?
- As a consumer would you have any concerns?
- Are there other factors or things you think need to be considered?

**OK let run the final poll – RUN POLL 2**

Now we are just going to change something again - <https://www.mvrdv.com/projects/134/pig-city>

Here are some images from a proposed vertical pig farming complex in the Netherlands

This is a bit scifi but this idea has been done on a smaller scale

- So does changing the species from chickens to pigs make a difference to your thinking
- If so why – why not

**Reflecting on these scenarios about fully automated livestock farming what are your thoughts on:**

- Increased use of automation and robotics across agriculture and food production?
- Is this inevitable – do we have a choice?
- What does it say about famers and our society if we no longer have direct contact with animals
- How do you think your community would respond to local agricultural production transitioning to AI or robotic production?

**ANY OTHER THOUGHTS YOU HAVE BEFORE WE GO**

**THANK YOU ALL VERY MUCH**
